# Safety and efficacy of resmetirom in the treatment of patients with non-alcoholic steatohepatitis and liver fibrosis: a systematic review and meta-analysis

**DOI:** 10.1097/MS9.0000000000002195

**Published:** 2024-05-22

**Authors:** Adarsh Raja, Raja Subhash Sagar, Sadia Saeed, Amna Zia ul haq, Owais Khan, Parshant Dileep Bhimani, Sandesh Raja, Fnu Deepak, Muhammad Ahmed, Muhammad Ashir Shafique, Muhammad Saqlain Mustafa, Muhammad Sohaib Asghar, Varsha Sharma

**Affiliations:** a Department of Internal Medicine, Shaheed Mohtarma Benazir Bhutto Medical College Lyari; b Department of Internal Medicine, Dow University of Health Sciences; c Department of Internal Medicine, Jinnah Sindh Medical University, Karachi; d Department of Internal Medicine, Liaquat University of Medical & Health Science, Jamshoro; e Department of Internal Medicine, Women Medical College Abbotabad, Abbottabad, Pakistan; f Department of Internal Medicine, Division of Nephrology and Hypertension, Mayo Clinic, Rochester, MN, USA; g Department of Internal Medicine, Nepal Medical College, Gokarneshwar, Nepal

**Keywords:** liver fibrosis, non-alcoholic steatohepatitis (NASH), placebo, resmetirom, thyroid hormone receptor-beta agonist

## Abstract

**Introduction:**

Non-alcoholic fatty liver disease (NAFLD), spanning from non-alcoholic steatohepatitis (NASH) to liver fibrosis, poses a global health challenge amid rising obesity and metabolic syndrome rates. Effective pharmacological treatments for NASH and liver fibrosis are limited.

**Objective:**

This study systematically reviews and meta-analyzes the safety and efficacy of resmetirom, a selective thyroid hormone receptor-β agonist, in NASH and liver fibrosis treatment. By analyzing data from clinical trials, we aim to offer evidence-based recommendations for resmetirom’s use in managing these conditions and identify avenues for future research.

**Methods:**

Electronic databases (PubMed, Scopus, Science Direct, Google Scholar, ClinicalTrials.gov, and Cochrane CENTRAL) were systematically searched, supplemented by manual screening of relevant sources. Only English-language randomized controlled trials were included. Data extraction, risk of bias assessment, pooled analyses, and meta-regression were performed.

**Results:**

Three randomized controlled trials involving 2231 participants were analyzed. Resmetirom demonstrated significant reductions in hepatic fat fraction [standardized mean difference (SMD) −4.61, 95% CI −6.77 to −2.44, *P* < 0.0001], NASH resolution without worsening fibrosis [risk ratio (RR) 2.51, 95% CI 1.74–3.64, *P* = 0.00001), and liver fibrosis improvement (RR 2.31, 95% CI 1.20–4.44, *P* = 0.01). Secondary outcomes showed significant improvements in lipid profiles, liver enzymes, and NASH biomarkers with resmetirom treatment. Meta-regression revealed associations between covariates and primary outcomes.

**Conclusion:**

Resmetirom exhibits promising efficacy in reducing hepatic fat, improving NASH resolution, and ameliorating liver fibrosis with a favorable safety profile. Further research is warranted to validate findings and optimize therapeutic strategies for NASH and liver fibrosis management.

## Introduction

Highlights

- Resmetirom effectively reduces the liver fat content.
- Non-alcoholic steatohepatitis (NASH) resolution without worsening fibrosis is significantly improved with resmetirom treatment.
- Resmetirom shows notable improvement in liver fibrosis.
- Lipid profiles and liver enzymes show positive changes with Resmetirom treatment.
- Resmetirom demonstrates a favorable safety profile.

Non-alcoholic fatty liver disease (NAFLD) encompasses a spectrum of liver conditions ranging from simple steatosis to non-alcoholic steatohepatitis (NASH) and liver fibrosis, which can progress to cirrhosis and hepatocellular carcinoma^1,2^. NASH, characterized by hepatic steatosis, inflammation, and hepatocellular injury, has emerged as a leading cause of liver-related morbidity and mortality worldwide^3^. With the increasing prevalence of obesity, type 2 diabetes, and metabolic syndrome, NAFLD has become a significant public health concern, affecting ~25% of the global population^4,5^. The rise in NAFLD incidence is particularly alarming among pediatric populations, underscoring the need for early intervention strategies^6^. Beyond its hepatic manifestations, NAFLD is also associated with an increased risk of cardiovascular disease, type 2 diabetes, and chronic kidney disease, further exacerbating its clinical impact^7^. Emerging evidence suggests that genetic factors may play a role in the development and progression of NAFLD, highlighting the need for personalized approaches to disease management^8^. Additionally, environmental factors, including exposure to endocrine-disrupting chemicals and air pollution, have been implicated in the pathogenesis of NAFLD, presenting new avenues for research and intervention^9^.

Despite the growing prevalence and clinical impact of NASH and liver fibrosis, effective pharmacological treatments are currently limited^10^. Lifestyle modifications, including diet and exercise, remain the cornerstone of management, but many patients fail to achieve adequate disease control with these interventions alone^11,12^. Thus, there is an urgent need for novel therapeutic strategies that can target the underlying pathophysiology of NASH and halt the progression of liver fibrosis.

Resmetirom, a selective thyroid hormone receptor-β agonist, has emerged as a promising candidate for treating NASH and liver fibrosis^13^. Preclinical studies have demonstrated that resmetirom modulates hepatic gene expression in lipid metabolism, inflammation, and fibrogenesis, improving liver histology and function^14^. Moreover, early clinical trials have shown encouraging results, with resmetirom demonstrating safety and efficacy in reducing hepatic steatosis, inflammation, and fibrosis in patients with NASH^14^.

However, despite these promising findings, the safety and efficacy of resmetirom in the treatment of NASH and liver fibrosis still need to be understood^14^. Existing studies have reported conflicting results, with some suggesting significant improvements in liver histology and function, while others have failed to demonstrate meaningful clinical benefits^14^. Furthermore, the safety profile of resmetirom, particularly in terms of cardiovascular outcomes and thyroid function, is an area that requires further investigation. While studies have shown that resveratrol, a compound with similar properties to resmetirom, is generally well-tolerated and has potential cardiovascular benefits^15^.

To address these knowledge gaps, we conducted a systematic review and meta-analysis of published literature to evaluate resmetirom’s safety and efficacy in treating patients with NASH and liver fibrosis. By synthesizing data from relevant clinical trials and observational studies, we aim to provide clinicians and policymakers with evidence-based recommendations for using resmetirom to manage NASH and liver fibrosis. Additionally, our study seeks to identify potential areas for future research and development, ultimately improving outcomes for patients with this debilitating condition.

## Methods

This systemic review and meta-analysis was registered on PROSPERO (CRD42024516985)^16^ and is conducted according to the Preferred Reporting Items for Systematic Reviews and Meta-Analyses (PRISMA) guidelines^17^, Supplemental Digital Content 1, http://links.lww.com/MS9/A492.

### Data sources and search strategy

A comprehensive search was carried out by using electronic databases like PubMed, Scopus, Science Direct, Google Scholar, ClinicalTrials.gov and Cochrane Central Register of Controlled Trials (CENTRAL) using MeSH terms like “Resmetirom”, “NASH” and “NAFLD”. Studies were manually searched as well (through journals, websites and snowballing by scrutinizing the bibliographies of Review articles) and in order to find grey literature, conference proceedings and presentations were also searched. Articles published in English language were included while there is no other restriction of time or sample size. The detailed search strategies used in these databases are provided in Supplementary Table 1, Supplemental Digital Content 2, http://links.lww.com/MS9/A493.

### Data synthesis

All studies extracted from the systematic search were subsequently exported into EndNote Reference Library (Version X7.5; Clarivate Analytics) to remove duplicates and streamline the screening process. To ensure relevance based on our inclusion criteria, two reviewers (M.A. and M.A.S.) independently evaluated the articles first based on the title and abstract, and later on an elaborate full-text review. Any disagreements were discussed with a third reviewer (A.R.).

All studies conforming with the following criteria were selected for inclusion; (i) Studies published in English language (ii) studies reporting outcomes of interest (iii) Published Randomized Control Trials, that why ongoing trials NCT05500222 and NCT03900429 were excluded. All letters, case reports, abstracts, reviews and extension studies were excluded from inclusion criteria.

### Data extraction

Two independent investigators (S.R. and M.A.) extracted data from the included studies into a Microsoft Excel (Microsoft Corporation) sheet. The outcome of interest of this systemic review and meta-analysis were (I) change from baseline in hepatic fat fraction by magnetic resonance imaging proton density fat fraction (MRI-PDFF) at 52 weeks (ii) lipid profile changes (low density lipoprotein (LDL) cholesterol, Apolipoprotein B, Triglyceride, Lipoprotein A) (iii) liver enzymes levels [alanine aminotransferase (ALT), aspartate aminotransferase (AST), γ-glutamyl transferase (GGT)] (iv) NASH Biomarkers (Adiponectin, CK-18/M30, reverse T3) (v) adverse events.

Whereas these baseline characteristics were also extracted like mean age, BMI, MRI-PDFF, fibrosis stage of the included population.

### Risk of bias and quality assessment

Exclusive inclusion of Randomized control trials enabled our analyzers (S.R.) to use the Cochrane Risk of Bias Tool for Randomized Controlled Trials (RoB-2)^18^, to evaluate the quality of included randomized controlled trials (RCTs). The studies were evaluated according to their randomization process, deviations from intended interventions, missing outcome data, measurement of the outcome, and selection bias within reported results. All studies were comprehensively screened and subsequently rated as “low risk”, “moderate risk”, or “high risk” of bias. RoB assessment was performed by two independent reviewers (M.A.S and M.S.M) with any discrepancy resolved by (F.D.). The AMSTAR 2, Supplemental Digital Content 3, http://links.lww.com/MS9/A494 tool is utilized for assessing methodological quality, ensuring that the conclusions and findings are rooted in the utmost quality evidence^19^.

### Statistical analysis

Review Manager software (version 5.4.1; Copenhagen: The Nordic Cochrane Centre, The Cochrane Collaboration, 2020) was used for analysis. For dichotomous outcomes, the risk ratio (RR) was used, whereas the standardized mean difference (SMD) was used to compare differences in outcomes reporting continuous data. The 95% CI were calculated for all outcomes. A *P* value of less than or equal to 0.05 was considered statistically significant for all outcomes. Heterogeneity was assessed utilizing the Higgins I^2^ test^20^. Values exceeding I^2^ = 50% were considered significantly heterogeneous, requiring additional examination through sensitivity analyses employing the leave-one-out method.

## Results

### Study characteristics

**Figure 1:**
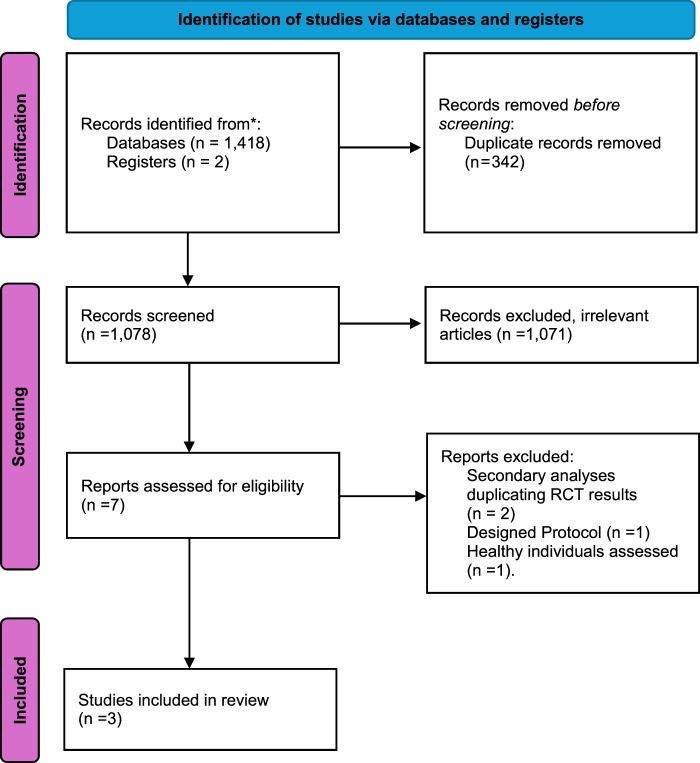
PRISMA Flow Chart. PRISMA, Preferred Reporting Items for Systematic Reviews and Meta-Analyses; RCT, randomized controlled trial.

**Figure 2:**
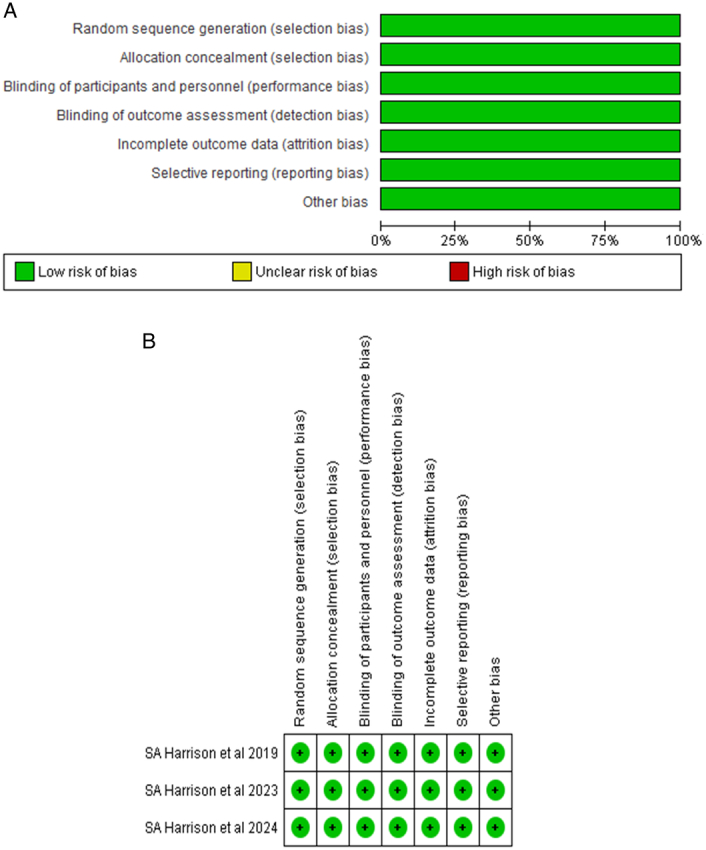
(A) Risk of bias graph. (B) Risk of bias summary.

During our literature screening, we used our search string to find 1418 total studies. However, only 3 studies^14,21,22^ met our inclusion criteria and were deemed eligible for use in our meta-analysis; these studies were all RCTs. A synopsis of our screening is provided in the PRISMA flowchart Fig. 1. The adult population was included in the sample size range, and both males and females were included in the sample size. There were 2231 people who took part in all. Different doses of resmetirom were used in our study intervention: two RCTS received 100 mg of resmetirom orally once daily, while three RCTS received 80 mg. Nonetheless, the placebo-treated control group was the same in every study. Table 1and Table 2 provides details regarding all studies and patients baseline characteristics. The details of the risk of bias assessment are given in Figure 2A, B and Supplementary Table 2, Supplemental Digital Content 2, http://links.lww.com/MS9/A493, and all the included studies were rated as being of high quality.

**Table 1:** General characteristics of included studies table.

						Sample size (*n*)	
Studies	Clinical trial number	Study design	Study duration	Country	Dose of resmetirom (mg)	Resmetirom	Placebo
Harrison *et al*. 2024^22^	NCT03900429	Multinational, randomized, double blinded, placebo-controlled phase 3 trial	March 28, 2019 till January 2024	USA, 245 sites	80 mg	322	321
					100 mg	323	
Harrison *et al*. 2023^21^	NCT04197479	Randomized, double blinded, placebo-controlled phase 3 trial	December 16, 2019 till January 6, 2023	USA	100 mg OL	171	318
					100 mg DB	324	
					80 mg DB	327	
Harrison *et al*. 2019^19^	NCT02912260	Multicentre, randomized, double blinded, placebo-controlled phase 2 trial	September 2016 till April 2018	USA, 25 sites	80 mg	84	41

DB, double blind; mg, milligram; NCT, National Clinical Trial; OL, open label.

**Table 2:** Patient baseline characteristics table.

	Sex male/female		Age (Mean, SD)		BMI		Fibrosis 4 index		MRI-PDFF, % fat fraction		LDL-cholesterol levels		
Studies	Resmetirom	Placebo	Resmetirom	Placebo	Resmetirom	Placebo	Resmetirom	Placebo	Resmetirom	Placebo	Resmetirom	Placebo	Follow-up time period
Harrison *et al*. 2024^22^	140/182	143/187	55.9±11.5	57.1±10.5	35.5±6.4	35.3±6.5	1.4±0.7	1.4±0.7	18.2±6.8	17.8±6.8	106.6±37.4	106.8±41.4	52 weeks
	141/182		57.0±10.8		36.2±7.4		1.5±0.7		17.2±6.6		103.0±36.8		
Harrison *et al*. 2023^21^	55/116	150/168	55.6±11.5	55.7±12.1	36.1±6.3	35.2±5.8	1.0±0.6	1.10±0.5	17.9±7.1	17.8±6.9	115.2±41.0	106.8±37.2	24 weeks
	147/177		55.9±11.7		35.4±6.4		1.0±0.4		18.1±7.3		109.1±36.4		
	145/182		56.2±11.7		35.3±5.9		1.0±0.5		17.7±6.7		111.7±37.6		
Harrison *et al*. 2019^19^	38/46	24/17	51·8±10·4	47·3±11·7	35·8±6·2	33·6±5·8	—	—	20·2±6·8	19·6±8·2	111·3±30·4	116·9±30·0	36 weeks

LDL, low density lipoprotein; MRI-PDFF, magnetic resonance imaging proton density fat fraction

### Primary outcomes

**Figure 3:**
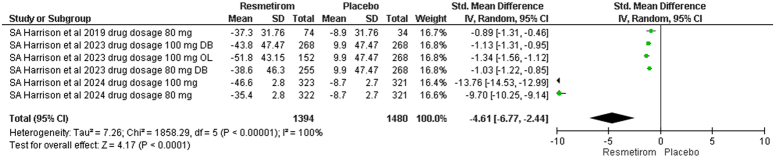
Forest plot of change from baseline in hepatic fat fraction by MRI-PDFF at 52 weeks.

The primary outcomes of our study, as defined by PICO criteria, change from baseline in hepatic fat fraction by MRI-PDFF at 52 weeks and clinical endpoints outcomes. Our main aim in performing this meta-analysis was to find out whether Resmetirom is superior in hepatic fat fraction by MRI-PDFF, NASH resolution without worsening fibrosis and improvement in liver fibrosis. During our study assessment, RCTS reported Change from Baseline in MRF-PDFF at week 52. Our analysis showed a significant trend towards fat reduction in the group that was administered with resmetirom as compared to the placebo group. The SMD was −4.61 (95% CI −6.77, −2.44: *P*<0.0001, I^2^=100%) Fig. 3. These results show that there are significant trends toward the use of resmetirom with lowering hepatic fat as compared to placebo. There was also significant heterogeneity in the outcome.

**Figure 4:**
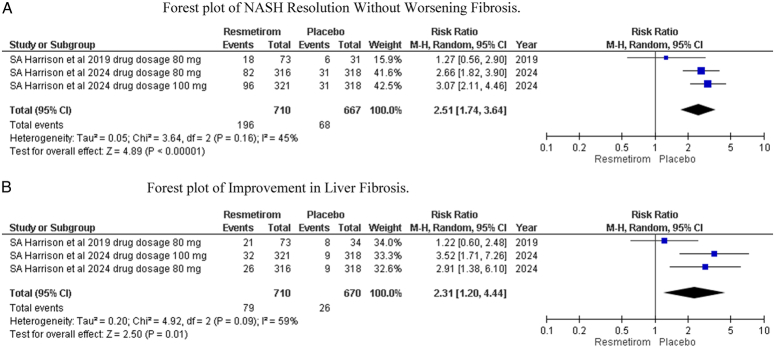
(A) Forest plot of non-alcoholic steatohepatitis resolution without worsening fibrosis. (B) Forest plot of improvement in liver fibrosis.

During the analysis of our second primary outcomes, that is NASH resolution without worsening fibrosis and improvement in liver fibrosis, we found 2 studies^14,22^ reporting positive results for second primary outcomes. The combined risk ratio for NASH resolution without worsening fibrosis, 2 studies showed a significant trend towards resmetirom, which was our intervention group, at 2.51 (95% CI 1.74, 3.64: *P*=0.00001, I^2^= 45%) Fig. 4A. The combined risk ratio for improvement in liver fibrosis, 2 studies showed a significant trend towards resmetirom, which was our intervention group, at 2.31 (95% CI 1.20, 4.44: *P*=0.01, I^2^= 59%) Fig. 4B.

### Secondary outcomes

Various secondary outcomes were found in the range of RCTs we used for our meta-analysis. Most of them were significant enough, while others were not that significant. The forest plot of all the secondary outcomes is attached in the supplementary file, Supplemental Digital Content 2, http://links.lww.com/MS9/A493. A brief review of all the secondary outcomes encountered is given below.

#### Treatment emergent adverse events

Three studies were included in the analysis^14,21,22^, which measured the spectrum of treatment emergent adverse events (TEAEs) encompassing mild, moderate, severe, and serious occurrences. Pooled analysis of the studies shows that the risk ratio using the random effect model was found to be 1.03 (95% CI 0.92, 1.15: *P*= 0.63, I^2^=0%) for mild TEAEs, 1.08 (95% CI 1.01, 1.16: *P*= 0.02, I^2^=0%) for moderate TEAEs, 0.87 (95% CI 0.72, 1.07: *P*= 0.18, I^2^=0%) for severe TEAEs and 1.00 (95% CI 0.79, 1.26: *P*= 1.00, I^2^=0%) for serious TEAEs. The statistical analysis shows that there is a significant difference between resmetirom and placebo in moderate TEAEs with *p* value of 0.02; however, the overall analysis effects between resmetirom and placebo shows no difference 1.05 (95% CI 0.99, 1.11: *P*= 0.10, I^2^=0%) Supplementary Figure 1, Supplemental Digital Content 2, http://links.lww.com/MS9/A493.

#### Total complications

Three studies were included in the analysis^14,21,22^, which measured total complications. Pooled analysis of the three studies shows that there were less complication events in placebo group *n*= 1352; than resmetirom group *n*= 1681, the risk ratio using the random effect model was found to be 1.22 (95% CI 1.02, 1.46 *P*=0.03, I^2^= 84%). The statistical analysis shows that there is a significant difference between Resmetirom and placebo. Supplementary Figure 2, Supplemental Digital Content 2, http://links.lww.com/MS9/A493.

#### Lipid profile changes

Three studies were included in the analysis^14,21,22^, which measured Change from Baseline at 24 weeks in Low Density Lipoprotein cholesterol, Apolipoprotein B, Triglyceride and Lipoprotein A levels. Pooled analysis of the studies shows that the SMD using the random effect model was found to be −3.28 (95% CI −5.19, 1.37: *P*=0.0008, I^2^=100% for Low Density Lipoprotein cholesterol; Supplementary Figure 3A, Supplemental Digital Content 2, http://links.lww.com/MS9/A493, −5.31 (95% CI −7.48, −3.15: *P*=0.00001, I^2^=100% for Apolipoprotein B; Supplementary Figure 3B, Supplemental Digital Content 2, http://links.lww.com/MS9/A493, −1.92 (95% CI −3.62, −0.22: *P*=0.03, I^2^=100% for Triglyceride; Supplementary Figure 3C, Supplemental Digital Content 2, http://links.lww.com/MS9/A493 and −3.19 (95% CI −5.43, −0.95: *P*=0.005, I^2^=100% for Lipoprotein A; Supplementary Figure 3D, Supplemental Digital Content 2, http://links.lww.com/MS9/A493. The statistical analysis shows that there is a significant difference between resmetirom and placebo in lipid profile at 24 weeks, which favors resmetirom group.

#### Liver enzymes

Three studies were included in the analysis^14,21,22^, which measured Change from Baseline at 48–52 weeks in ALT, AST and GGT levels. Pooled analysis of the studies shows that the SMD using the random effect model was found to be −2.29 (95% CI −4.29, −0.28: *P*=0.03, I^2^=100% for ALT; Supplementary Figure 4A, Supplemental Digital Content 2, http://links.lww.com/MS9/A493, −2.13 (95% CI −3.78, −0.47: *P*=0.01, I^2^=100% for AST; Supplementary Figure 4B, Supplemental Digital Content 2, http://links.lww.com/MS9/A493, −2.13 (95% CI −3.76, −0.49: *P*=0.01, I^2^=100% for GGT; Supplementary Figure 4C, Supplemental Digital Content 2, http://links.lww.com/MS9/A493. The statistical analysis shows that there is a significant difference between resmetirom and placebo in liver enzymes at 48–52 weeks which favors resmetirom group.

#### NASH biomarkers

Two studies were included in the analysis^14,21^, which measured Change from Baseline at 52 weeks in CK-18/M30, Adiponectin and Reverse T3 levels. Pooled analysis of the studies shows that the SMD using the random effect model was found to be −0.31 (95% CI −0.47, −0.16: *P* <0.0001, I^2^=49% for CK-18/M30; Supplementary Figure 5A, Supplemental Digital Content 2, http://links.lww.com/MS9/A493, 0.37 (95% CI 0.17, 0.57: *P*=0.0003, I^2^=61% for Adiponectin; Supplementary Figure 5B, Supplemental Digital Content 2, http://links.lww.com/MS9/A493, −0.77 (95% CI −0.87, −0.67: *P* <0.00001, I^2^=0% for Reverse T3; Supplementary Figure 5C, Supplemental Digital Content 2, http://links.lww.com/MS9/A493. The statistical analysis shows that there is a significant difference between resmetirom and placebo in NASH Biomarkers at 52 weeks which favors resmetirom group for CK-18/M30, Reverse T3 and favors placebo group for Adiponectin.

*Meta-regression*: We assessed mean age, male sex, BMI, and comorbidities such as hypertension, diabetes mellitus, dyslipidemia, hypothyroidism, and Atherosclerotic cardiovascular disease (ASCVD) as a potential covariate affecting the effect size on our primary outcome, change in MRI-PDFF. All the estimates of effects showed a significant association between each of these covariates and MRI-PDFF at 52 weeks except mean age, male sex %, BMI and hypothyroidism. The results are as follows: mean age, Coeff: 1.5896, *P*=0.4551; male sex, Coeff: −1.7056, *P*=0.4662; BMI, Coeff: −0.5726, *P*=0.9837; diabetes mellitus, Coeff: 1.3005, *P*=0.0000; hypertension Coeff: −4.6845, *P*=0.0033; dyslipidemia Coeff: 1.2959, *P*=0.0000; hypothyroidism, Coeff: 1.0600, *P*=0.1795; ASCVD, Coeff: 10.3316, *P*=0.0057 (Supplementary figures 6A-6H, Supplemental Digital Content 2, http://links.lww.com/MS9/A493) Assessment of Heterogeneity

*Assessment of heterogeneity*: To ensure accurate pooled estimates, robustness, and prevention of disproportionate impacts, a sensitivity analysis was conducted. This analysis included leaving out studies, excluding those with small or large sample sizes and outliers, particularly for primary and secondary outcomes with I2 values of 50% or greater and heterogeneity p values less than 0.05. Despite these efforts, the sensitivity analysis, including the leave-one-out analysis, did not reveal any significant changes.

## Discussion

The U.S. Food and Drug Administration (FDA) has released draft guidelines for clinical trials focusing on NASH drugs for patients with NASH and compensated cirrhosis. Currently, only a few drugs have gained FDA approval for NASH treatment, with most still undergoing clinical trials. NASH is a heterogeneous disease influenced by genetic determinants, environmental factors, and comorbidities, all contributing to fibrosis progression in specific individuals. Fibrosis emerges as a crucial predictor of clinical outcomes, and cardiovascular issues are the leading cause of death in NASH patients^23^. In this meta-analysis, Resmetirom has shown promise as an effective and well-tolerated treatment for NASH and liver fibrosis, demonstrating significant reductions in hepatic fat fraction, improved liver enzyme levels, and positive effects on non-invasive markers of liver fibrogenesis and stiffness.

The utilization of Resmetirom leads to a significant reduction in fat, attributed to the activation of THR-β, a nuclear hormone receptor crucial for regulating impaired metabolic pathways in NAFLD and NASH^24^. A pooled analysis of three studies reveals a noteworthy reduction in ALT and AST levels with Resmetirom treatment compared to placebo, indicating improved liver function, reduced hepatic inflammation, and overall enhanced liver health. Additionally, Resmetirom demonstrates a significant impact on NASH biomarkers, suggesting efficacy in modulating key underlying causes through the modulation of lipophagy, mitophagy, and mitogenesis within hepatocytes^25^. Navigating the diagnostic landscape of NASH proves challenging, often leading to late-stage identification utilizing invasive techniques like liver biopsy. This delayed diagnosis has earned NASH the ominous moniker of the “Silent Killer.” The absence of a cost-effective and minimally invasive diagnostic test further complicates estimating disease prevalence^26^. The histological basis for a NASH diagnosis lies in the NAFLD activity score (NAS), encompassing steatosis, inflammation, and hepatocyte ballooning as a measure of disease activity. Once diagnosed, disease progression is gauged by the NASH Clinical Research Network (CRN) fibrosis score, ranging from 0 (none) to 4 (cirrhosis). Regulatory bodies, such as the FDA and EMA, advocate focusing drug development on non-cirrhotic NASH with liver fibrosis (fibrosis score greater than 1 but less than 4), addressing stages deemed to have the greatest need^26,27^. Notably, a group of experts has initiated a debate on the terminology for NAFLD, proposing metabolic-associated fatty liver disease (MAFLD) as a more fitting term to capture the disease’s heterogeneity, adding nuance to its understanding^27^. The mortality associated with NASH is substantial, leading to a global burden of NASH-related liver cancer deaths, reaching 34.7 thousand in 2019. The mortality rate for NASH-related liver cancer has increased by 7.86% in individuals older than 55 years from 2010 to 2019, reaching 2.11 per 100 000 population in 2019. Additionally, deaths due to NASH-related liver cancer were reported as 4.93 thousand for individuals aged 20–54 years old^28^. High cardiovascular mortality is observed alongside liver cancer. Furthermore, individuals with NASH, predominantly those with diabetes, face elevated cardiovascular risk and mortality^29,30^. Earlier phase 1 studies showed that daily doses of resmetirom between 50 mg and 200 mg resulted in statistically significant lowering of atherogenic lipids^31^. However, the observed preference for a placebo in Adiponectin levels suggests a complex interplay between Resmetirom’s effects on lipid metabolism and adiponectin regulation, warranting further investigation. Overall, this meta-analysis underscores the multifaceted benefits of Resmetirom in NASH treatment, spanning fat reduction, fibrosis resolution, cardiovascular health improvement, and modulation of key biomarkers, with implications for future therapeutic strategies^32^. Achieving a weight loss of at least 5% is crucial for hepatic steatosis regression, while 7% and 10% weight loss can, respectively, improve inflammation and fibrosis. Despite the pivotal role of lifestyle changes, it proves challenging for NAFLD patients, with fewer than 10% achieving success in weight loss through this approach. While lifestyle modification remains central, there is currently no widely accepted treatment for NASH^33^.

Obesity is a common risk factor associated with NASH and NAFLD. Anti-obesity drugs are being studied in clinical trials, such as Orlistat, an oral gastrointestinal lipase inhibitor with a weight-loss effect. Orlistat reduces fat absorption and prevents triglycerides from entering the liver. In a randomized, double-blind, placebo-controlled trial, weight loss was comparable between patients using Orlistat and placebo, while ultrasound detected a more significant improvement of fatty liver in patients using Orlistat for 24 weeks than in the control group^34^. However, Harrison et al. conducted a randomized controlled trial (RCT) using pathological score as the endpoint and revealed that for patients with NASH, a 36-week treatment with orlistat was not superior to a change in lifestyle in terms of weight loss, improvement of fatty liver degeneration, or serum transaminase^35^. Numerous pharmacological agents, including thyroid hormone receptor-beta (THR-β) selective agonists like TZDs, have been studied. Omega-3 fatty acids have been shown to reduce oxidative stress, lipotoxicity, and inflammation in patients with NASH^36^. However, UDCA at 13–15 mg/kg body weight in biopsy-proven NASH patients lacks efficacy compared to placebo and is not recommended^37^. Early studies involving metformin demonstrated improvements in insulin resistance, liver chemistries, and a modest reduction in hepatic steatosis. However, two subsequent meta-analyses examining the use of metformin in NASH found no discernible benefit, leading to its current non-recommendation for NASH treatment^38,39^. Furthermore, a study indicated that pioglitazone displayed comparable efficacy in both diabetic and non-diabetic NAFLD patients, enhancing histopathology, liver enzymes, and insulin resistance without additional adverse effects^40^. Another study suggested that serum ALT levels and histological parameters improved in NASH patients treated with TZDs. Notably, there’s a proposition that pioglitazone could potentially reverse fibrosis in NASH^41^. Additionally, a study demonstrated the cost-effectiveness of resmetirom through a Markov model, indicating that resmetirom had a higher probability of being cost-effective compared to placebo (incremental cost-effectiveness ratio = $74,018) and was dominant versus OCA (being both less costly and more effective)^32^.

Our meta-analysis confirms the scientific rigor and reliability of the included studies but also highlights notable limitations, particularly concerning biochemical, histological parameters and particularly in addressing heterogeneity. The scarcity of relevant RCTs, with only three included, highlights the challenge of obtaining RCTs. Heterogeneity among the studies’ outcomes raises concerns about publication bias, compounded by limited access to unpublished results. Furthermore, the fact that the three included trials were authored by the same group may have contributed to higher heterogeneity in the results. This emphasizes the importance of diversifying study sources to mitigate potential biases and enhance the robustness of findings. Additionally, the analysis overlooks the impact of short follow-up periods on long-term insights and omits a crucial cost-effectiveness assessment, hindering generalizability. Detailed baseline characteristics were lacking, limiting insights into potential influencing factors. The absence of subgroup analysis, attributed to the incorporation of studies involving two dosage variants (80 mg and 100 mg resmetirom), adds complexity to our comprehension of potential dose-related effects. These considerations underscore the need for cautious interpretation and future research refinement.

## Conclusion

Resmetirom exhibited a notable trend in reducing hepatic fat fraction compared to placebo, as evidenced by MRI-PDFF at 52 weeks. Moreover, significant trends towards NASH resolution without worsening fibrosis, along with improvement in liver fibrosis and secondary outcomes, were observed in the Resmetirom group, indicating its potential to arrest or even reverse disease progression. Importantly, Resmetirom demonstrated a favorable safety profile, with no statistically significant differences in overall treatment-related adverse events compared to placebo. Further, long-term follow-up and large-scale sample trials are imperative to refine and validate our findings comprehensively.

## Ethics approval and consent to participate

Not applicable.

## Consent for publication

Not applicable.

## Sources of funding

The authors received no extramural funding for the study.

## Author contribution

All authors made a significant contribution to the work reported, whether that is in the conception, study design, execution, acquisition of data, analysis and interpretation, or in all these areas; took part in drafting, revising or critically reviewing the article; gave final approval of the version to be published; have agreed on the journal to which the article has been submitted; and agree to be accountable for all aspects of the work.

## Conflicts of interest disclosure

The authors declare that they have no known competing financial interests or personal relationships that could have appeared to influence the work reported in this paper.

## Research registration unique identifying number (UIN)

This systemic review and meta-analysis was registered on PROSPERO (CRD42024516985).

## Guarantor

Varsha Sharma.

## Availability of data and materials statement

The dataset supporting the conclusions of this article are included in this article.

## Provenance and peer review

Not commissioned, externally peer-reviewed.

## Supplementary Material

SUPPLEMENTARY MATERIAL
