# Supplementary material for: Safety and efficacy of resmetirom in the treatment of patients with non-alcoholic steatohepatitis and liver fibrosis: a systematic review and meta-analysis
Source: Ann Med Surg (Lond). 2024 May 22;86(7):4130–8. doi: 10.1097/MS9.0000000000002195 (PMC11230798; doi:10.1097/MS9.0000000000002195)
Supplement: SUPPLEMENTARY MATERIAL [file ms9-86-4130-s002.docx]

**Supplementary Material**

**Supplementary Table 1.** Detailed search strategy used in each database.

| **Databases** | **Search String** | **Results Obtained** |
| --- | --- | --- |
| PubMed | (Resmetirom OR MGL-3196) AND (NASH OR non-alcoholic steatohepatitis OR non-alcoholic fatty liver disease OR NAFLD) | 42 |
| Scopus | (Resmetirom OR MGL-3196) AND (NASH OR non-alcoholic steatohepatitis OR non-alcoholic fatty liver disease OR NAFLD) | 150 |
| Cochrane Library | (Resmetirom OR MGL-3196) AND (NASH OR non-alcoholic steatohepatitis OR non-alcoholic fatty liver disease OR NAFLD) | 51 |
| ScienceDirect | (Resmetirom OR MGL-3196) AND (NASH OR non-alcoholic steatohepatitis OR non-alcoholic fatty liver disease OR NAFLD) | 193 |
| Google Scholar | (Resmetirom OR MGL-3196) AND (NASH OR non-alcoholic steatohepatitis OR non-alcoholic fatty liver disease OR NAFLD) | 982 |

**Supplementary Table 2.** Risk of Bias Assessment Table

|  | Cochrane Risk-of-Bias Tool | | |
| --- | --- | --- | --- |
|  | Bias | Risk of bias | Author judgement |
| SA Harrison et al 2019 | Random sequence generation (selection bias) | Low Risk | Randomization was adequately performed using a computer-based system, minimizing the risk of biased allocation to interventions. |
|  | Allocation concealment (selection bias) | Low Risk | The allocation concealment process was not explicitly described, but since the study was double-blinded and used identical placebo, it's likely that allocation concealment was adequately implemented, minimizing the risk of biased allocation to interventions. |
|  | Blinding of participants and personnel (performance bias) | Low Risk | Participants, investigators, and site personnel were effectively blinded to treatment assignment throughout the study, reducing the risk of performance bias. |
|  | Blinding of outcome assessment (detection bias) | Low Risk | Outcome assessors were blinded to treatment allocation, reducing the risk of detection bias. |
|  | Incomplete outcome data (attrition bias) | Low Risk | Incomplete outcome data were appropriately handled, minimizing the risk of attrition bias. |
|  | Selective reporting (reporting bias) | Low Risk | There is no evidence of selective outcome reporting, reducing the risk of reporting bias. |
|  | Other bias | Low Risk | No other biases were identified in the study that could significantly impact the results. |
| SA Harrison et al 2023 | Random sequence generation (selection bias) | Low Risk | Patients were randomized to different treatment arms in a 1:1:1 ratio using an interactive voice and web response system, indicating adequate random sequence generation and minimizing biased allocation to intervention |
|  | Allocation concealment (selection bias) | Low Risk | Although the allocation concealment process was not explicitly described, the trial was double-blind, and patients and personnel were unaware of individual patient identification and treatment assignments. Therefore, it's likely that allocation concealment was effectively implemented, minimizing biased allocation to interventions. |
|  | Blinding of participants and personnel (performance bias) | Low Risk | The trial was double-blind, meaning both participants and personnel were unaware of the treatment assignments, reducing the risk of performance bias. |
|  | Blinding of outcome assessment (detection bias) | Low Risk | Outcome assessors were blinded to treatment allocation, and assessments such as MRI-PDFF, MRE, and FibroScans were performed by trained personnel who were unaware of the patients' treatment groups, reducing the risk of detection bias. |
|  | Incomplete outcome data (attrition bias) | Low Risk | The primary analysis included a substantial number of patients across the treatment arms, and missing data were appropriately addressed in the analysis. Patients with missing data were accounted for, and sensitivity analyses were conducted to assess the impact of missing data on the outcomes. This approach minimizes the risk of attrition bias and enhances the reliability of the study findings. |
|  | Selective reporting (reporting bias) | Low Risk | All primary and secondary endpoints were pre-specified in the trial protocol, and the results for each were reported in the manuscript, reducing the risk of reporting bias |
|  | Other bias | Low Risk | No other biases were identified that could significantly impact the results. The trial adhered to ethical guidelines and regulatory requirements. |
| SA Harrison et al 2024 | Random sequence generation (selection bias) | Low Risk | Patients were randomly assigned in a 1:1:1 ratio to receive different doses of resmetirom or placebo using an interactive Web-response system, indicating adequate random sequence generation and minimizing biased allocation to interventions. |
|  | Allocation concealment (selection bias) | Low Risk | The trial was double-blind, and the allocation concealment process was not explicitly described. However, since the trial remained blinded to individual patient identification and trial-group assignments, it's likely that allocation concealment was effectively implemented, minimizing biased allocation to interventions. |
|  | Blinding of participants and personnel (performance bias) | Low Risk | The trial was double-blind, meaning both participants and personnel were unaware of the treatment assignments, reducing the risk of performance bias |
|  | Blinding of outcome assessment (detection bias) | Low Risk | Outcome assessors were blinded to treatment allocation, as biopsy specimens were assessed centrally by two independent expert pathologists who were unaware of the trial-group assignments, reducing the risk of detection bias. |
|  | Incomplete outcome data (attrition bias) | Low Risk | The primary analysis population consisted of 966 patients, and missing data were handled appropriately. Patients with missing biopsies were considered to have not had a response, minimizing the risk of attrition bias |
|  | Selective reporting (reporting bias) | Low Risk | All primary and secondary endpoints were pre-specified in the trial protocol, and the results for each were reported in the manuscript, reducing the risk of reporting bias |
|  | Other bias | Low Risk | No other biases were identified in the study that could significantly impact the results. The trial was conducted in accordance with ethical guidelines and regulatory requirements. |

**Supplementary Figure 1.** Forest Plot of Adverse Event


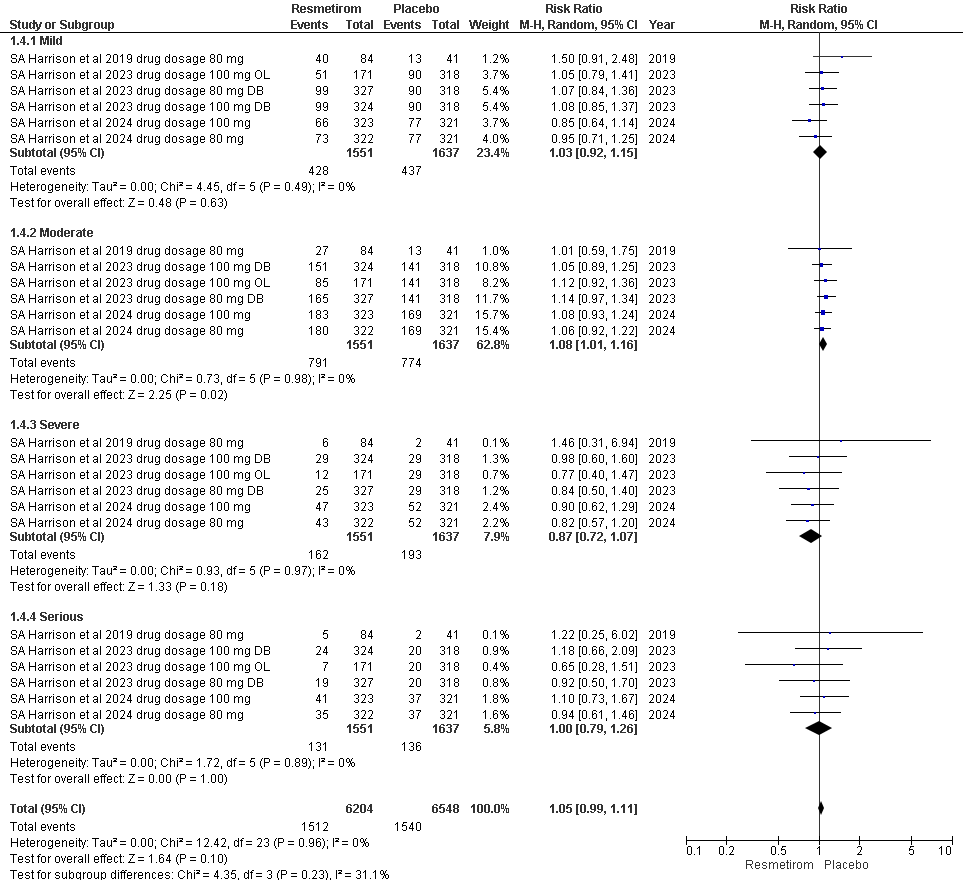


**Supplementary Figure 2.** Forest Plot of Total Complications


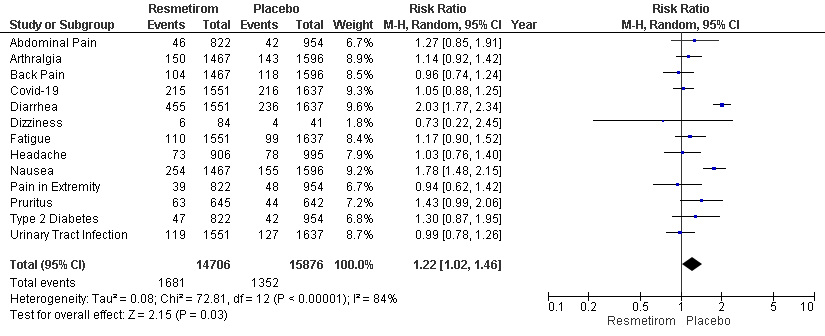


**Supplementary Figure 3A.** Forest Plot of Change from Baseline in LDL cholesterol level at 24 weeks


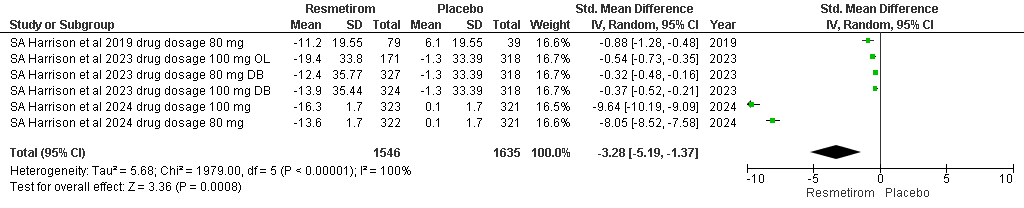


**Supplementary Figure 3B.** Forest Plot Change from Baseline in Apolipoprotein B level at 24 weeks


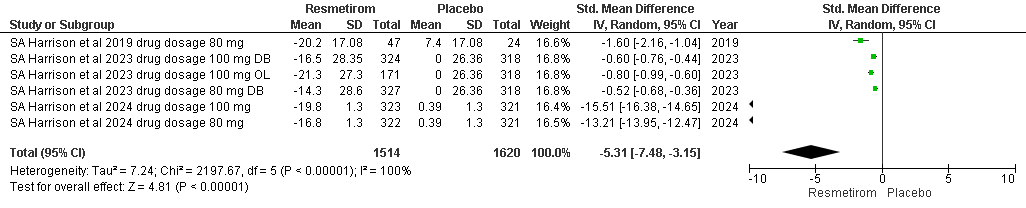


**Supplementary Figure 3C.** Forest Plot Change from Baseline in Triglyceride level at 24 weeks


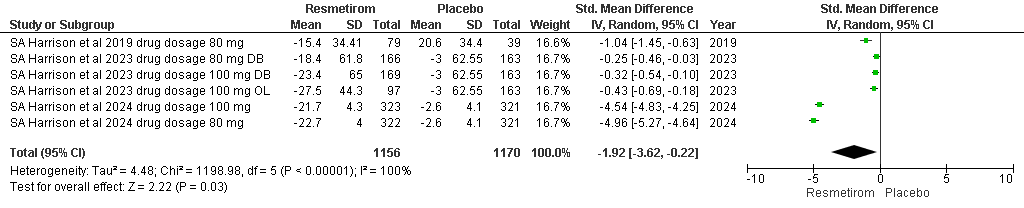


**Supplementary Figure 3D.** Forest Plot Change from Baseline in Lipoprotein A level at 24 weeks


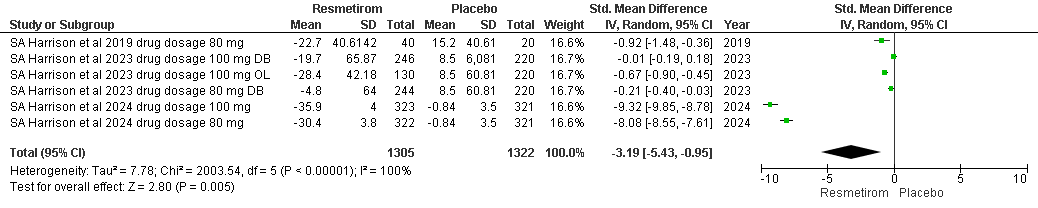


**Supplementary Figure 4A.** Forest Plot Change from Baseline in Alanine aminotransferase level at 48-54 weeks


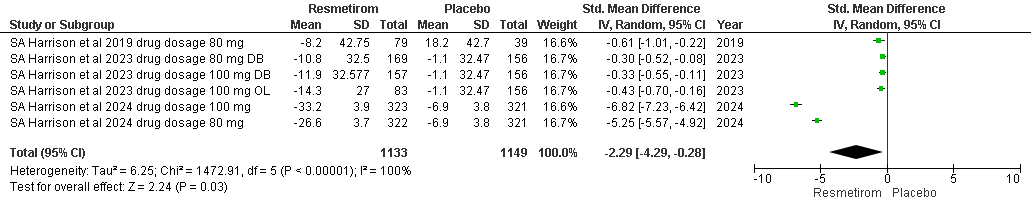


**Supplementary Figure 4B.** Forest Plot Change from Baseline in Aspartate aminotransferase level at 48-54 weeks


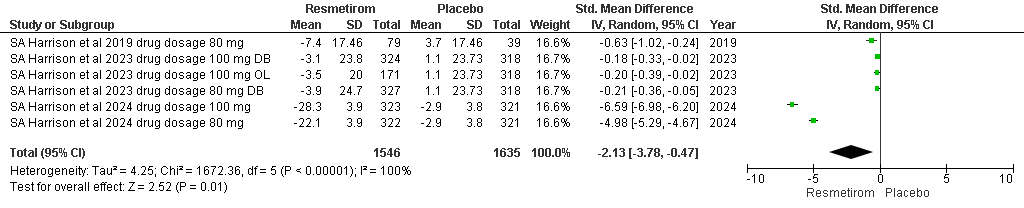


**Supplementary Figure 4C.** Forest Plot Change from Baseline in γ-Glutamyl transferase level at 48-54 weeks


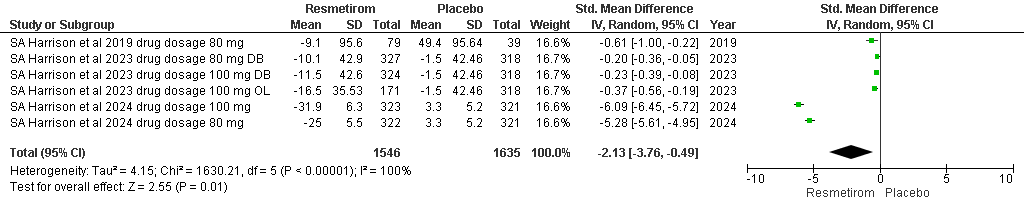


**Supplementary Figure 5A.** Forest Plot Change from Baseline in CK-18/M30, Ul −1 at 52 weeks


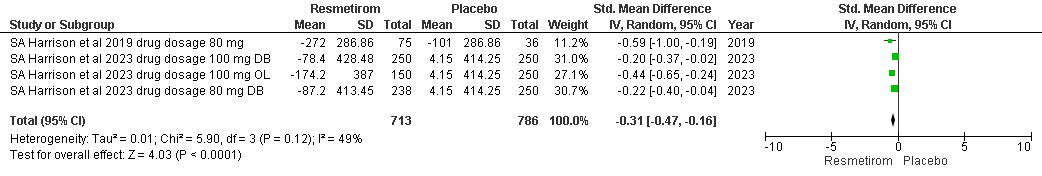


**Supplementary Figure 5B.** Forest Plot Change from Baseline in Adiponectin, μgml−1 at 52 weeks


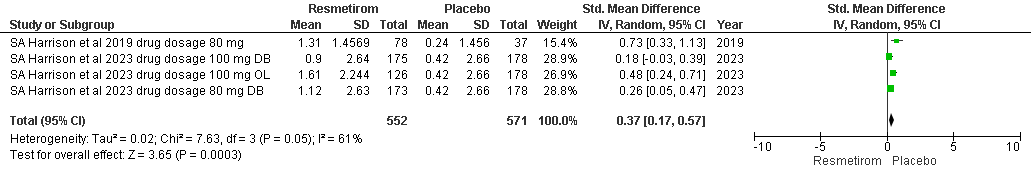


**Supplementary Figure 5C.** Forest Plot Change from Baseline in Reverse T3, ngdl−1 at 52 weeks


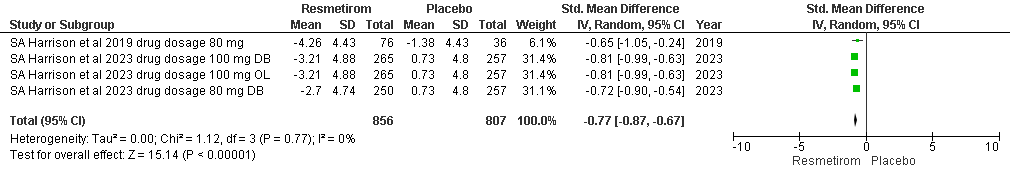


**Supplementary Figure 6A.** Regression plot for mean age

**Supplementary Figure 6B.** Regression plot for male sex%


**Supplementary Figure 6C.** Regression plot for BMI


**Supplementary Figure 6D.** Regression plot for diabetes mellitus


 **Supplementary Figure 6E.** Regression plot for hypertension


**Supplementary Figure 6F.** Regression plot for dyslipidemia

 **Supplementary Figure 6G.** Regression plot for hypothyroidism

**Supplementary Figure 6H.** Regression plot for ASCVD
